# Analysis of *Gzmb^Cre^* as a Model System for Gene Deletion in the Natural Killer Cell Lineage

**DOI:** 10.1371/journal.pone.0125211

**Published:** 2015-04-29

**Authors:** Yiying Xu, Cesar Evaristo, Maria-Luisa Alegre, Sandeep Gurbuxani, Barbara L. Kee

**Affiliations:** 1 Committee on Molecular Pathogenesis and Molecular Medicine, University of Chicago, Chicago, Illinois, United States of America; 2 Committee on Immunology, University of Chicago, Chicago, Illinois, United States of America; 3 Committee on Cancer Biology, University of Chicago, Chicago, Illinois, United States of America; 4 Department of Pathology, University of Chicago, Chicago, Illinois, United States of America; 5 Department of Medicine, Section of Rhuematology, University of Chicago, Chicago, Illinois, United States of America; Oklahoma Medical Research Foundation, UNITED STATES

## Abstract

The analysis of gene function in mature and activated natural killer cells has been hampered by the lack of model systems for Cre-mediated recombination in these cells. Here we have investigated the utility of *Gzmb^Cre^* for recombination of loxp sequences in these cells predicated on the observation that *Gzmb* mRNA is highly expressed in mature and activated natural killer cells. Using two different reporter strains we determined that gene function could be investigated in mature natural killer cells after *Gzmb^Cre^* mediated recombination in vitro in conditions that lead to natural killer cell activation such as in the cytokine combination of interleukin 2 and interleukin 12. We demonstrated the utility of this model by creating *Gzmb^Cre^*;*Rosa26^IKKbca^* mice in which Cre-mediated recombination resulted in expression of constitutively active IKKβ, which results in activation of the NFκB transcription factor. In vivo and in vitro activation of IKKβ in natural killer cells revealed that constitutive activation of this pathway leads to natural killer cell hyper-activation and altered morphology. As a caveat to the use of *Gzmb^Cre^* we found that this transgene can lead to recombination in all hematopoietic cells the extent of which varies with the particular loxp flanked allele under investigation. We conclude that *Gzmb^Cre^* can be used under some conditions to investigate gene function in mature and activated natural killer cells.

## Introduction

Natural killer (NK) cells are lymphocytes that function at the intersection of innate and adaptive immunity and they are promising targets for cancer immunotherapy [1]. They recognize virus-infected, stressed, or cancerous cells through multiple germ line encoded activating and inhibitory receptors [2]. When an imbalance in these signaling inputs occurs that favors activating over inhibitory receptor signaling, NK cells rapidly produce inflammatory cytokines including interferon (IFN) γ and tumor necrosis factor (TNF) α and undergo degranulation releasing perforin and granzymes to kill associated target cells [3]. NK cells can also be activated by dendritic cell (DC) derived inflammatory cytokines such as interleukin (IL)12 and IL18 and they can alter DC function through numerous mechanisms thereby augmenting or limiting the adaptive immune response [4]. NK cells are considered a component of the innate immune system due to their basal primed effector state, which allows for rapid responses prior to engagement of the adaptive immune response. However, recent studies have revealed that NK cells, like adaptive immune cells, can display characteristics of memory cells including a heightened response to secondary challenge and antigen specificity [5,6,7].

Our understanding of the molecular mechanisms controlling NK cell function is limited, particularly when compared to our understanding of adaptive immune system cells. One reason for this under-appreciation is that model systems in which genes can be specifically deleted from, or expressed in, NK cells are not widely available. Indeed, until recently, the only way to test gene function in mature (m) NK cells was through targeted disruption of a gene in the germ line or in all hematopoietic cells using Cre recombinase expressing transgenes that delete in hematopoietic stem cells such as *Vav* ^*Cre*^ or *Tie2* ^*Cre*^ [8,9]. These models have the obvious caveat that genes that are required for development of the multipotent progenitors of early NK lineage cells cannot be tested in mNK cells. For example, the functions of the nuclear factor (NF) κB family have been investigated in NK cells using germ line deletion of two inhibitors of this transcription factor, IκBα and IκBε whose deletion results in constitutive activity of NFκB. In *Iκbα* ^*-/-*^ *;Iκbε* ^*-/-*^ mice, NK cell development arrests at the immature (i) NK cell stage suggesting that constitutive activation of NFκB is lethal at a stage prior to the development of mNK cells [10]. In contrast, human patients with an inactivating mutation in the IKKγg/NEMO kinase, which functions in a complex that promotes NFκB activation by phosphoryating IκB proteins and targeting them for ubiquitination and degradation, develop mNK cells that show limited cytotoxic function [11]. It remains unclear whether this reduced cytotoxic capacity is due to requirements for NFκB in mNK cells or during earlier stages of NK cell development, where a lack of functional NFκB may have impaired acquisition of cytolytic competence. Consequently, the significance of NFκB activation in mNK cells has not been directly evaluated.

Recently, mouse strains were described that produce Cre under the control of the *Ncr1* promoter [12] or the entire *Ncr1* locus [13], which encodes for the activating NK cell receptor NKp46 [14,15]. In these mice, Cre-mediated recombination initiates during the iNK cell stage, prior to the generation of mNK cells but downstream of the most immature NK cell progenitors (NKP) [16]. *Ncr1* is expressed primarily in NK cells but is also expressed in subsets of T cells and innate lymphoid cells, requiring that both populations be considered in phenotype interpretation of mice in which a gene is deleted using *Ncr1* ^*Cre*^ [13]. While the *Ncr1* ^*Cre*^ strains are highly useful for investigating gene function in NK cells, the field could benefit from additional Cre-producing strains that delete at later stages of NK cell development or after NK cell activation.

Here we report that the *Gzmb* ^*Cre*^ transgenic mouse can be used to delete genes in activated mNK cells. *Gzmb* ^*Cre*^ has been used extensively for studies of effector and memory CD8 T cells because it promotes Cre-mediated recombination in CD8 T cells at the height of the effector response [17]. We found that *Cre* mRNA was expressed at a low level throughout the hematopoietic system of *Gzmb* ^*Cre*^ mice and that Cre-mediated recombination could detected in many cell types. Nonetheless, activated mNK cells from *Gzmb* ^*Cre*^ mice strongly upregulated *Cre* mRNA and efficiently recombined loxp-flanked sequences. We propose that *Gzmb* ^*Cre*^ can be used for loxp-dependent deletion or activation of genes in activated mNK cells in vitro or after transplantation in vivo, after which NK cell function can be assessed using multiple assays. We demonstrated the utility of *Gzmb* ^*Cre*^ by crossing this allele into *Rosa26* ^*IKKbca*^ mice, which produce a constitutively active form of the IKKβ protein (IKKβ-CA) after Cre-mediated recombination. The *Gzmb* ^*Cre*^;*Rosa26* ^*IKKbca*^ genotype allowed us to investigate the consequences of NFκB activation in mNK cells and revealed that constitutive activation of IKKβ led to hyperactivation and morphological alterations.

## Methods and Materials

### Mice

This study was carried out in strict accordance with the recommendations in the Guide for the Care and Use of Laboratory Animals of the National Institutes of Health. The protocol was approved by the Institutional Animal Care and Use Committee of the University of Chicago (Assurance # A3523-01) under the Animal Care and Use Protocol # 71116. Animals were sacrificed using CO_2_ asphyxiation followed by cervical disclocation. The *Rosa26* ^*IKKbca*^ (C57BL/6- *Gt(Rosa)26Sor* ^*tm1(Ikbkb)/Rsky/*^J) [18], *Rosa26* ^*EYFP*^ (B6.129X1- *Gt(Rosa)26Sor* ^*tm1(EYFP)Cos/*^ *J*) [19] and *Rosa26* ^*ACTB-tdTomato*, *-EGFP*^ (C57BL/6-*Gt(Rosa)26Sor* ^*tm4(ACTB-tdTomato*, *-EGFP)Luo/*^J) mice [20] were purchased from Jackson Laboratories. *Gzmb* ^*Cre*^ mice on a C57Bl/6 background were provided by Dr. E. John Wherry and have been described previously [17].

### Flow Cytometry

Cells were stained with fluorochrome- or biotin-labeled antibodies for 30 min on ice. The following antibodies conjugated to FITC, PE, PerCp-Cy5.5, PerCP-ef710, PeCy7, APC, APC-ef780, Pacific Blue, or Brilliant Violet 421 were purchased from eBiosciences or BD Biosciences: CD3ε, CD4, CD8α, CD19, Ter119, CD122, NK1.1, NKp46, DX5, CD69, CD11b, CD25, Klrg1, IL7Rα, Sca1, cKit, CD43, B220, and Gr1. Propidium iodide was used to exclude dead cells. Cells were acquired on a FACS Canto, LSRII, or Fortessa or sorted with a FACS ARIAII and analyzed with FLOWjo. Through this study mNK cells were identified using a lineage cocktail containing CD19, CD3, CD4, CD8, and Ter119 antibodies and propidium iodide to exclude dead cells. NK cells were either Lin-CD122+NK1.1^+^ or Lin-CD122+NKp46/NK1.1+ and DX5^+^. NKP were defined as Lin-CD122^+^NK1.1^-^.

### NK cell cultures and activation

Primary mNK cells were isolated from the spleen of mice on a FACSAria and culture in vitro in conditions that were approved by the Institutional Biosafety Committee at the University of Chicago. The NK cells were cultured in Opti-MEM supplemented with 10% FBS, 80μM 2-mercaptoethanol, 100U/ml penicillin, 100μg/ml streptomycin, and 29.2mg/ml glutamine, IL2 (1000 IU/ml, NIH AIDS Reagents program) with or without IL12 (3.3ng/ml, Peprotech). In vivo, NK cells were activated by an intraperitoneal injection of 150μg (150 μl of a 1 mg/ml solution) of poly(I:C) and evaluated 72 hrs post injection by flow cytometry.

### QPCR

RNA was purified from FACS sorted cells using the RNAeasy micro kit (QIAGEN) and reverse-transcribed with Superscript III (Invitrogen). QPCR was performed with gene-specific primers in an iCycler (BioRad) using the iQ SYBR Green Supermix (BioRad) as recommended by the manufacture. *Hprt*, amplified in the same RNA sample, was used to normalize gene expression. *Hprt*-66F, 5′-ACCTCTCGAAGTGTTGGATA-3′; *Hprt*-66R, 5′-CAA CAA CAA ACT TGT CTG GA-3′; Cre for, 5’- CGT ACT GAC GGT GGG AGA AT-3’; Cre rev 5’-CCC GGC AAA ACA GGT AGT TA-3’; *Gzmb* for 5’-ACA GAA GGA TCG GGA GTG TG -3’; *Gzmb* rev 5’-CTA TGC CTG CAG CCA CTT TT -3’

### Statistical Analysis

Standard t-tests were used to determine statistical significance using the GraphPad software program.

## Results and Discussion

### Variegated expression of YFP in mNK cells from *Gzmb* ^*Cre*^ *;Rosa26* ^*EYFP*^ mice

**Fig 1:**
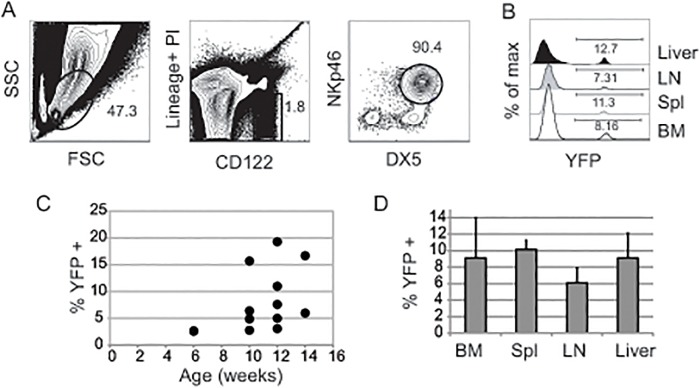
*Gzmb* ^*Cre*^;*Rosa26* ^*EYFP*^ mice have a low frequency of YFP^+^ mNK cells. (A) Representative FACS histograms showing the gating strategy for mNK cells in *Gzmb* ^*Cre*^;*Rosa26* ^*EYFP*^ mice. Lymphoid cells were gated by FSC and SSC (left panel) and then Lineage/PI negative cells that express CD122 (center plot) were selected and examined for expression of NKp46 and DX5. In some experiments mNK cells were Lineage/PI negative CD122^+^NK1.1^+^ as shown in Fig 3A. (B) The frequency of mNK cells expressing EYFP in a typical *Gzmb* ^*Cre*^;*Rosa26* ^*EYFP*^ mouse bone marrow (BM), spleen (spl), lymph node (LN) and liver. (C) Scatter plots showing frequency of YFP^+^ mNK cells in the bone marrow of multiple *Gzmb* ^*Cre*^;*Rosa26* ^*EYFP*^ mice at different ages. Each circle represents one mouse. (D) The average number of YFP^+^ mNK cells is shown for each of the indicated tissues in mice between 6 and 12 weeks of age. Error bars = standard deviation. n > 5 for each tissue. The difference in average YFP^+^ mNK cell number between different tissues was not statistically significant.

*Gzmb* mRNA is expressed in mNK cells and increases upon NK cell activation leading us to hypothesize that *Gzmb* ^*Cre*^ might be useful for recombination of loxp-flanked sequences in these cells. To test this hypothesis we crossed *Gzmb* ^*Cre*^ mice, which produce Cre under the control of a fragment of the human *Gzmb* promoter [17], to multiple reporter mouse strains. We examined *Gzmb* ^*Cre*^;*Rosa26* ^*EYFP*^ mice, in which the *Rosa26* locus expresses enhanced yellow fluorescent protein (YFP) only after Cre-mediated recombination and deletion of a loxp-flanked STOP translation sequence that is upstream of the YFP coding sequence [19]. Flow cytometry analysis revealed that only a portion of the Lin^-^CD122^+^NKp46^+^DX5^+^ mNK cells from the bone marrow of these mice expressed YFP (Fig 1A and 1B). The frequency of YFP positive cells in any given mouse was similar among mNK cells isolated from the bone marrow, spleen, liver or lymph node (Fig 1B). The frequency of YFP^+^ mNK cells varied between 3 and 20% in the bone marrow of mice as old as 12 weeks of age but there was no significant difference in the frequency of YFP^+^ cells in any of the tissues examined (Fig 1D). Therefore, at least a subset of mNK cells or their precursors expressed Cre from the *Gzmb* ^*Cre*^ allele.

### Activation-dependent recombination of *Rosa26* ^*EYFP*^ by *Gzmb* ^*Cre*^

**Fig 2:**
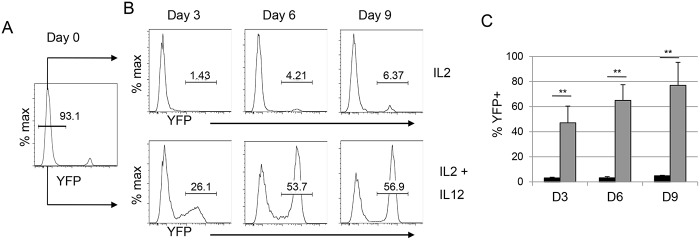
In vitro activation promotes Cre-mediated recombination in *Gzmb* ^*Cre*^ *;*Rosa26^*EYFP*^ mNK cells. (A) YFP^-^ mNK cells were isolated from the spleen of *Gzmb* ^*Cre*^;*Rosa26* ^*EYFP*^ mice by flow cytometric sorting and they were cultured in (B) IL2 alone (upper panels) or IL2 + IL12 (lower panels) for 3, 6 or 9 days prior to analysis by flow cytometry for YFP expression. (C) The average frequency of YFP^+^ mNK cells on day 3, 6, and 9 after in vitro culture in IL2 (black) or IL2 + IL12 (grey). Error bars = standard deviation. n>5, ** p < 0.01.

We hypothesize that *Gzmb* ^*Cre*^ may be more highly expressed in activated mNK cells than in resting mNK cells because *Gzmb* mRNA increases with NK cell activation. To test this hypothesis we isolated YFP negative mNK cells from *Gzmb* ^*Cre*^;*Rosa26* ^*EYFP*^ mice by cell sorting and cultured these cells in vitro in IL2 alone or IL2 + IL12 (Fig 2A and 2B). Mature NK cells cultured in IL2 proliferate and become primed but they are not fully activated [21]. A small fraction of IL2-stimulated YFP negative NK cells began to express YFP with approximately 6% of NK cells being YFP^+^ by day 9 of culture (Fig 2B and 2C). In contrast to IL2 alone, IL2 + IL12 results in NK cell activation including degranulation and production of IFNγ [21]. When cultured in IL2 + IL12 a greater frequency of mNK cells began to express YFP, reaching frequencies > 55% between 6 and 9 days after activation (Fig 2B and 2C). Our data demonstrated that *Gzmb* ^*Cre*^ recombines loxp-flanked sequences in a subset of mNK cells and that recombination could be augmented in vitro under conditions of activation.

### Reporter variation in *Gzmb* ^*Cre*^-mediated recombination

**Fig 3:**
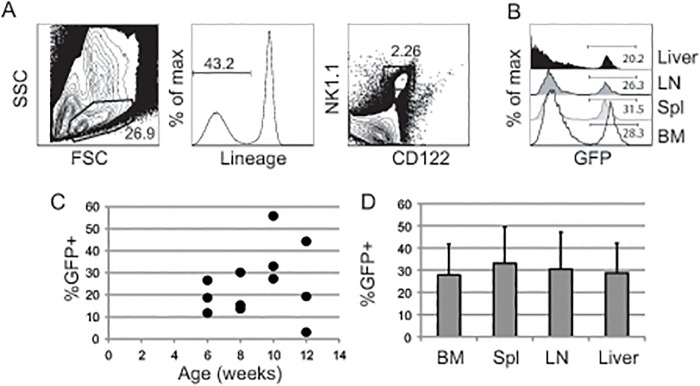
*Gzmb* ^*Cre*^;*Rosa26* ^*ACTB-tdTomato*, *-EGFP/+*^ mice have a low frequency of GFP^*+*^ mature NK cells. (A) Representative FACS histograms showing the gating strategy for mNK cells in *Gzmb* ^*Cre*^;*Rosa26* ^*ACTB-tdTomato*, *-EGFP/+*^ mice. Lymphoid cells were gated by FSC and SSC (left panel) and then Lineage negative, PI- cells (center plot) were selected and examined for expression of NK1.1 and CD122. (B) The frequency of mNK cells expressing GFP in a typical *Gzmb* ^*Cre*^;*Rosa26* ^*ACTB-tdTomato*, *-EGFP/+*^ mouse bone marrow (BM), spleen, lymph node (LN) and liver. The frequency of GFP^*+*^ cells is indicated. (C) Scatter plots showing the frequency of YFP^*+*^ mNK cells in the bone marrow of multiple *Gzmb* ^*Cre*^;*Rosa26* ^*ACTB-tdTomato*, *-EGFP/+*^ mice at different ages. Each circle represents one mouse. (D) The average number of GFP^*+*^ mNK cells is shown for each of the indicated tissues in mice between 6 and 12 weeks of age. Error bars = standard deviation. n > 7 for each tissue. The difference in average GFP^*+*^ mNK cell number between different tissues was not statistically significant.

It has been noted previously that reporter alleles can differ in their sensitivity to recombination by Cre and reporter assays have been developed to exploit engineered differences [22,23,24,25,26]. Therefore, we assessed the ability of *Gzmb* ^*Cre*^ to promote recombination of a second reporter construct. We chose the *Rosa26* ^*ACTB-tdTomato*, *-EGFP*^ reporter, which produces a transmembrane form of tomato red fluorescent protein (RFP) constitutively under the control of the actin promoter, which is inserted into the Rosa26 locus [20]. Cre-mediated recombination deletes the tomato red coding sequence and brings the sequence for enhanced green fluorescent protein (GFP) under the control of the actin promoter. In *Gzmb* ^*Cre*^;*Rosa26* ^*ACTB-tdTomato*, *-EGFP/+*^ mice we again observed that only a subset of mNK cells in the bone marrow, spleen, lymph node and liver expressed the GFP reporter (Fig 3A and 3B), and this frequency was highly variable in mice between 6 and 12 weeks of age (Fig 3C). The frequency of GFP^+^ cells was similar in different tissues from the same mouse. On average, a higher frequency of mNK cells from *Gzmb* ^*Cre*^;*Rosa26* ^*ACTB-tdTomato*, *-EGFP/+*^ mice showed evidence of Cre activity compared to those in *Gzmb* ^*Cre*^;*Rosa26* ^*EYFP*^ mice (Figs 3D and 1D, p<0.01 for all tissues).

**Fig 4:**
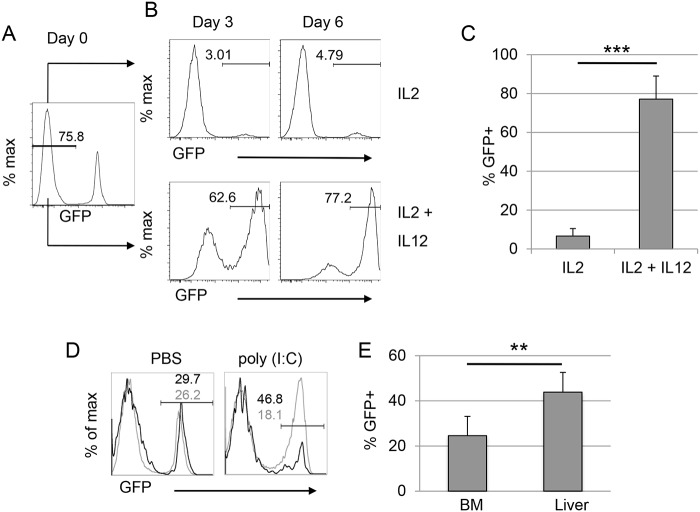
In vitro activation causes Cre-mediated recombination in *Gzmb* ^*Cre*^;*Rosa26* ^*ACTB-tdTomato*, *-EGFP/+*^ mature NK cells. (A) GFP^-^ mNK cells were isolated from the spleen of *Gzmb* ^*Cre*^;*Rosa26* ^*ACTB-tdTomato*, *-EGFP/+*^ mice by flow cytometric sorting and cultured in (B) IL2 alone (upper panels) or IL2 + IL12 (lower panels) for 3 or 6 days prior to analysis by flow cytometry for GFP expression. (C) The average frequency of GFP^+^ mNK cells on day 6 of culture for cells grown in IL2 or IL2 + IL12 is shown. n = 5, *** = p<0.001. (D) Mice were injected i.p. with 150 ug of poly(I:C) or PBS as control, and analyzed 3 days later. GFP^+^ cells in the bone marrow (black) and liver (grey) are shown from a representative PBS (left plot) or poly(I:C) (right plot) injected mouse. (E) The average number of GFP^+^ mNK cells in the bone marrow and liver of poly(I:C) treated mice is indicated. Error bars = standard error. n = 4, ** = p< 0.01.

We next tested the ability of *Gzmb* ^*Cre*^ to recombine the *Rosa26* ^*ACTB-tdTomato*, *-EGFP*^ reporter in vitro. When GFP^-^ mNK cells were cultured in IL2 alone, a low frequency of cells began to express GFP within 6 days (Fig 4A–4C). In contrast, greater than 60% of GFP^-^ mNK cells became GFP^+^ within 3 days after initiation of culture in IL2 + IL12 (Fig 4B). On average, close to 80% of mNK cells were GFP^+^ by day 6 of culture in IL2 + IL12 (Fig 4C). To activate NK cells in vivo we injected *Gzmb* ^*Cre*^;*Rosa26* ^*ACTB-tdTomato*, *-EGFP/+*^ mice with 150 ug poly(I:C) i.v. and compared the number of GFP^+^ mNK cells to those in mice injected with a comparable volume of PBS. However, we were not able to reliably determine whether poly(I:C) treatment increased the frequency of GFP^+^ cells because of the variability we observed in the frequency of GFP^+^ mNK cells between individual animals. Nonetheless, poly(I:C) treatment did result in an increased frequency of eGFP^+^ mNK cells in the liver as compared to the bone marrow of individual mice (Fig 3D and 3E). This enrichment of GFP^+^ cells in the liver was not observed in PBS treated *Gzmb* ^*Cre*^;*Rosa26* ^*ACTB-tdTomato*, *-EGFP/+*^ mice (Fig 3D and 3E). Therefore, we conclude that *Gzmb* ^*Cre*^ recombines loxp-flanked sequences in activated mNK cells, which are then enriched in the liver.

### *Gzmb* ^*Cre*^ is expressed broadly in the hematopoietic system

**Fig 5:**
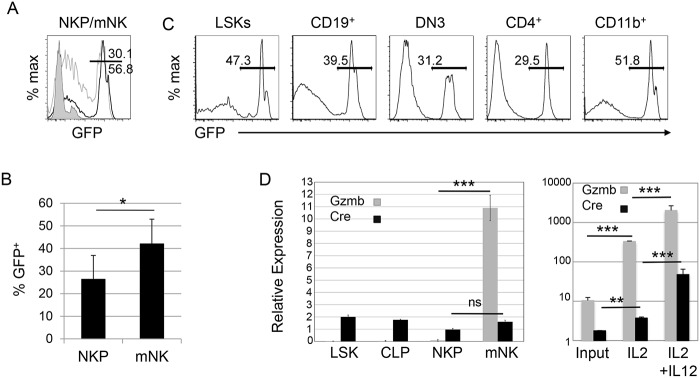
Detection of *Gzmb* ^*Cre*^-mediated recombination in multiple hematopoietic cell types. (A) Representative FACS plot showing GFP expression in bone marrow Lin/PI^-^CD122^+^NK1.1^-^ NKP (grey) and Lin/PI^-^CD122^+^NK1.1^+^ mNK cells (black) from an 8 week old *Gzmb* ^*Cre*^;*Rosa26* ^*ACTB-tdTomato*, *-EGFP/+*^ mouse. The shaded histogram is the isotype control. (B) The average number of GFP^+^ NKP and mNK cells from mice between 8 and 12 weeks of age. n = 5, * = p< 0.05 (C) eGFP expression in LSKs (Lin-SCA1^+^cKIT^+^), CD19^+^ bone marrow cells, DN3 cells, CD4^+^ thymocytes and CD11b^+^ bone marrow cells from mice at 12 weeks of age. One of three representative experiments is shown. (D) QPCR analysis of *Cre* (black) and *Gzmb* (grey) mRNA from LSKs, CLPs (Lin^-^CD127^+^cKIT^+^SCA1^+^Flt3^+^), NKPs, and mNK cells or from primary splenic NK cells or splenic NK cells cultured in IL2 or IL2 + IL12 for 6 days. mRNA expression was normalized to *Hprt* using the ΔCT method. n = 3, ** = p< 0.01, *** = p< 0.001.

To determine whether the *Gzmb* ^*Cre*^-mediated recombination observed in mNK cells in vivo was specific to mNK cells, we examined GFP expression in NK cell progenitors (NKP) using the *Rosa26* ^*ACTB-tdTomato*, *-EGFP*^ reporter. To our surprise, we found that GFP was expressed in these cells, albeit the average percentage of GFP^+^ cells was slightly lower than in the mNK cells from the same mouse (Fig 5A and 5B). This surprising observation indicated that Cre expression did not faithfully mimic endogenous *Gzmb* mRNA expression and prompted us to look more broadly among hematopoietic cells for *Gzmb* ^*Cre*^-directed recombination events. Remarkably, we found that CD19^+^ B cells, DN3 thymocytes, CD4^+^ thymocytes and CD11b^+^ macrophages all showed evidence of *Gzmb* ^*Cre*^-mediated recombination in this reporter strain (Fig 5C). Most notably, expression of GFP was also observed in multipotent hematopoietic progenitors including the Lin^-^SCA1^+^cKIT^+^ (LSK) population that contains hematopoietic stem cells (Fig 5C). An analysis *Cre* mRNA revealed that there was a low level of expression in LSKs and CLPs (Fig 5D). By contrast, *Gzmb* mRNA was not detected in LSKs or CLPs (Fig 5D). Notably, and in contrast to *Gzmb* mRNA, *Cre* mRNA did not increase in mNK cells compared to LSKs, explaining the low level of recombination in mNK cells (Fig 5D). However, in vitro activation of mNK cells resulted in a robust (>10-fold) induction of both *Gzmb* and *Cre* mRNA (Fig 5D). Our data demonstrate that *Gzmb* ^*Cre*^ had the potential to recombine loxp sequences in hematopoietic progenitors resulting in recombined alleles in many hematopoietic lineages. Nonetheless, recombination was increased specifically in activated mNK cells indicating that under some conditions *Gzmb* ^*Cre*^ can be used to investigate gene function in activated mNK cells.

### Constitutive activation of IKKβ in mNK cells resulted in an activated phenotype

**Fig 6:**
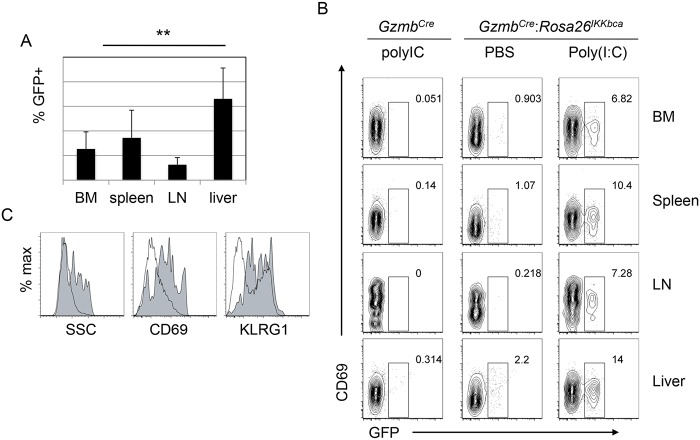
Constitutive IKKβ activation in mNK cells leads to characteristics of activation. (A) Bar graphs show the average frequency of GFP^+^ mNK cells in the bone marrow, spleen, lymph node and liver of *Gzmb* ^*Cre*^ *;Rosa26* ^*IKKbca*^ mice between 8 and 12 weeks of age. Error bars = standard error. n = 4, ** p<0.01. (B) *Gzmb* ^*Cre*^ *;Rosa26* ^*IKKbca*^ mice were injected with PBS or poly(I:C) (150 ug) and analyzed 72 hrs later by flow cytometry for expression of GFP and CD69 on mNK cells in the indicated tissues. *Gzmb* ^*Cre*^ mice injected with PBS were analyzed as a negative control for GFP. (C) Flow cytometric analysis of SSC, CD69 and KLRG1 from GFP^+^ (shaded histogram) and GFP^-^ (open histograms) *Gzmb* ^*Cre*^ *;Rosa26* ^*IKKbca*^ liver NK cells.

To further investigate the utility of *Gzmb* ^*Cre*^ for studies of gene function in mNK cells we examined *Gzmb* ^*Cre*^-mediated recombination of the *Rosa26* ^*IKKbca*^ transgene. This transgene encodes a constitutively active (CA) form of IKKβ, a kinase that phosphorylates IκB proteins leading to their ubiquitin-mediated degradation and consequently the activation of NFκB, knocked in to the *Rosa26* locus [18]. NFκB is activated in response to ligation of multiple activating NK cell receptors and inflammatory cytokines [27,28], and is proposed to be necessary for NK cell cytotoxicity [29]. Indeed, an inactivating mutation in IKKγ was found in multiple patients that had defective NK cell-mediated cytotoxic responses [11]. While NFκB is active at a low level in most NK cells, deregulation of its function through germ line mutation of IκBα and IκBε results a decline in mature NK cell numbers, arrested maturation and a failure to produce IFNγ [10]. Therefore, we tested whether constitutive activation of IKKβ, and consequently activation of NFκB, would lead to alterations in mNK cells. Notably, in vivo at steady state there were few GFP^+^ NK cells in the bone marrow, spleen, lymph nodes and liver of *Gzmb* ^*Cre*^;*Rosa26* ^*IKKbca*^ transgenic mice (Fig 6A). However, there were consistently more GFP^+^ cells in the liver than in the other tissues (Fig 6A). In contrast, *Gzmb* ^*Cre*^;*Rosa26* ^*ACTB-tdTomato*, *-EGFP/+*^ mice did not show a difference in GFP expression between bone marrow and liver mNK cells unless the mice were treated with poly(I:C) to activate the NK cells (Fig 4D). Therefore, the enrichment of GFP^+^ cells in the liver of *Gzmb* ^*Cre*^;*Rosa26* ^*IKKbca*^ transgenic mice at steady state is consistent with the hypothesis that the IKKβ-CA protein resulted in activation of the NK cells. In accordance with this hypothesis, the GFP^+^ cells in the liver of these mice were more granular than their GFP^-^ counterparts and they were enriched for expression of CD69 and KLRG1 (Fig 6B and 6C), which are markers of NK cell activation [30]. In contrast to the *Gzmb* ^*Cre*^;*Rosa26* ^*ACTB-tdTomato*, *-EGFP/+*^ mice, we did not detect GFP expression among CD19+ B lymphocytes or DN3 thymocytes (data not show). Injection of *Gzmb* ^*Cre*^;*Rosa26* ^*IKKbca*^ transgenic mice with poly(I:C) lead to a consistent but not significant increase in the number of GFP^+^ cells (Fig 6B). Therefore, mNK cells with constitutively active IKKβ can be detected in vivo and these cells show evidence of activation.

**Fig 7:**
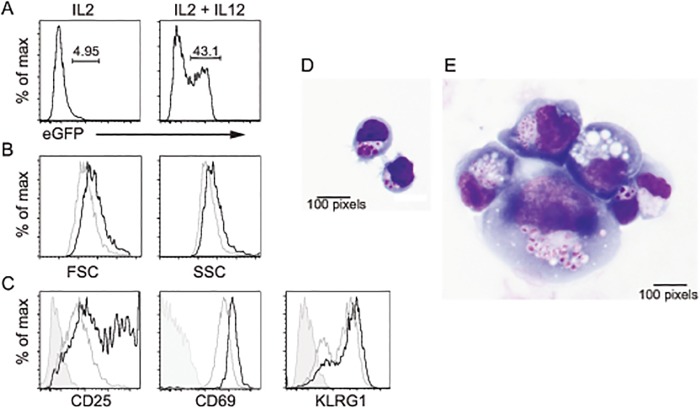
Constitutive IKKβ activation leads to NK cell hyper-activation and altered morphology. (A) GFP^-^ mNK cells from the spleen of *Gzmb* ^*Cre*^ *;Rosa26* ^*IKKbca*^ mice were isolated by cell sorting and cultured in IL2 or IL2 + IL12 for 5 days prior to analysis of GFP expression by flow cytometry. One representative experiment of 3 is shown. (B) FSC and SSC analysis of the GFP^+^ (black) and GFP^-^ (grey) cells from the day 5 IL2 + IL12 cultures. (C) Expression of CD25, CD69, and KLRG1 on the GFP^+^ (black) and GFP^-^ (grey) cells from the day 5 IL2 + IL12 cultures. The shaded grey histogram represents the isotype control staining for GFP^+^ cells. (D) Giemsa stain for GFP^-^ and (E) GFP^+^ cells 5 days after transfer of cells from IL2 + IL12 cultures to IL2 alone. One of 3 similar experiments is shown; 100 pixels = 10 μm, 400X.

To gain further insight into the consequences of IKKβ activation in mNK cells, we examined mNK cells after in vitro recombination of the *Rosa26* ^*IKKbca*^ transgene. As we observed with the *Rosa26* reporter alleles, in vitro culture of GFP^-^ *Gzmb* ^*Cre*^ *;Rosa26* ^*IKKbca*^ mNK cells in IL2 promoted weak recombination of the *Gzmb* ^*Cre*^;*Rosa26* ^*IKKbca*^ allele (Fig 7A). However, a substantial frequency of GFP^-^ *Gzmb* ^*Cre*^ *;Rosa26* ^*IKKbca*^ mNK cells became GFP^+^ after a 5 day culture in IL2 + IL12 (Fig 7A). IL2 + IL12 causes NK cell activation, however, the IKKβ-CA expressing mNK cells cultured under these conditions were even larger and more granular than their GFP^-^ counterparts (Fig 7B), indicating that these cells were hyperactivated. Indeed, the IKKβ-CA expressing cells also had very high surface expression of the activation markers CD25, CD69, and KLRG1 (Fig 7C). When transferred from IL2 + IL12 to IL2 alone, to allow the cells to return to conditions that are not activating, the GFP^+^ cells became irregular in appearance and adherent to the plastic dish making quantification difficult. On Giemsa stained preparations, the GFP^+^ cells were larger and showed large coarse granules when compared to the GFP^-^ cells (Fig 7D and 7E). These data led us to conclude that constitutive activation of IKKβ resulted in NK cell hyperactivation as measured by increased size and granularity and the high level of expression of activation markers, changes in adhesive properties, and altered morphologic characteristics. Therefore, while activation of NFκB is necessary for NK cell function, constitutive activation of NFκB could lead to chronic activation as well as functional and morphologic changes that could prevent their accumulation.

## Discussion

The *Gzmb* ^*Cre*^ mouse model has been used for studies of gene function in activated or memory CD8 T cells [17,31]. Since *Gzmb* is also expressed in NK cells we investigated the utility of this model for gene deletion in mNK cells. We found that *Gzmb* ^*Cre*^ can be used to investigate gene function in activated mNK cells, particularly in vitro. However, this Cre-deleter strain showed considerable variability with respect to the extent of deletion in mNK cells and in other hematopoietic cell types including hematopoietic stem cells and multipotent progenitors. On the low end of the spectrum a few percent of mNK cells and other cell types showed evidence of Cre-mediated recombination. In contrast, one of the reporters we utilized showed that greater than 50% of most hematopoietic cells had undergone recombination. These studies reveal the potential heterogeneity in detecting Cre-mediated recombination by different reporter alleles revealing that these reporters may not faithfully recapitulate recombination frequencies at an allele of interest. Moreover, our study also indicated that gene deletion with *Gzmb* ^*Cre*^ can be heterogeneous and can initiate as early as the hematopoietic stem cell in some strains of mice. Despite these caveats we were able to demonstrate the utility of this model for Cre mediated recombination in mNK cells in vitro and in vivo.

While our study raises some caveats with respect to the utility of *Gzmb* ^*Cre*^, we found that it could be used to investigate the function of genes that are directly linked to a reporter gene such as GFP. As an example, we examined the consequences of expressing constitutively active IKKβ in mNK cells. IKKβ is one of the two kinase subunits of the IKK complex, which phosphorylates the IκB proteins that hold multiple NFκB subunits in an inactive state in the cytoplasm [32]. Constitutive activation of IKKβ results in phosphorylation of IκB proteins resulting in their targeted degradation by the ubiquitin-proteasome system and nuclear translocation of NFκB transcription factors. We found that IKKβ-CA^+^ cells induced by *Gzmb* ^*Cre*^ could be detected at a low frequency in the absence of intentional NK cell activation and were more frequently found in the liver as compared to the bone marrow, spleen and lymph nodes. Therefore, constitutive activation of NFκB may influence the migration or tissue retention patterns of NK cells. IKKβ-CA mNK cells were more granular than their IKKβ-CA negative counterparts and showed higher expression of CD69, a marker associated with NK cell activation [33]. In vitro, the cytokine combination of IL2 + IL12 induced a subset of mNK cells to express the IKKβ-CA transgene and these cells could be maintained in vitro in IL2. Interestingly, IKKβ-CA^+^ mNK cells that survived for extended times in vitro showed a morphological change becoming extremely large and having an extensive granular network with large vacuoles. This phenotype was not observed when GFP^-^ cells were analyzed from the same starting sample or when YFP^+^ cells from *Gzmb* ^*Cre*^;*Rosa26* ^*EYFP*^ mice were tracked in vitro (data not shown). Therefore, constitutive express of IKKβ-CA results in hyperactivation of mNK cells and morphological changes. In vivo, the *Rosa26* ^*IKKbca*^ transgene could be expressed in other hematopoietic cells that might influence the fate of NK cells. However, the hyperactivation of mNK cells expressing IKKβ-CA appears to be an intrinsic consequence of IKKβ-CA expression in NK cells because it was observed in in vitro cultures of purified mNK cells that underwent Cre mediated recombination after the initiation of culture. Taken together, our results demonstrate that the *Gzmb* ^*Cre*^ transgenic mouse can be used for studies of gene function in activated mNK cells.
